# Supplementary material for: Amphiphysin AoRvs167-Mediated Membrane Curvature Facilitates Trap Formation, Endocytosis, and Stress Resistance in Arthrobotrys oligospora
Source: Pathogens. 2022 Aug 31;11(9):997. doi: 10.3390/pathogens11090997 (PMC9501185; doi:10.3390/pathogens11090997)
Supplement: Supplementary file 1 [file pathogens-11-00997-s001.zip › Supplementary Materials.pdf]

Supplementary Materials:

Supplementary Table S1. The functions of the proteins or the GO biological processes in Fig. 1B.

	Protein names	Functions or the GO biological processes
AoRVS167	G1X8J9(BAR domain-containing protein)	Other cellular component
AoRVS161	G1XUF7(BAR domain-containing protein)	Cytoskeleton organization, Other cellular component
AoACT	G1XT57(100% identity with actin in *A.oligospora*)	Involved in various types of cell motility
AoGYP5	G1XFI4(Rab-GAP TBC domain-containing protein)	Not clear
AoABP1	G1X1H9(Uncharacterized protein)	Cytoskeletal protein binding, Organelle
AoLAS17	G1XLT8(Uncharacterized protein)	Cytoskeletal protein binding, Cytoskeleton organization, Cytoskeleton
AoMYO5	G1XAX1(Myosin-1)	Cytoskeletal motor activity, Cytoskeletal protein binding, Hydrolase activity, Other cellular component ,Cytoskeleton

Supplementary Table S2. mRNA expression levels of *AoRvs161*(AOL_s00215g615) and *AoRvs167*(AOL_s00075g198).# represents time under ammonia-induced conditions.

Gene_id	readcount_Am3	readcount_NA	log2FoldChange	pval	padj	Gene Name	time#
22899063	34190.036	3642.630983	3.2305	0.00057992	0.002195	AOL_s00215g615	4h
22891708	42098.6662	4107.546204	3.3574	0.00084508	0.002999	AOL_s00075g198	
22899063	32300.31719	4347.087906	2.8934	1.44E-06	1.85E-05	AOL_s00215g615	24h
22891708	22335.86279	4907.783583	2.1862	0.00012848	0.000856	AOL_s00075g198	
22899063	37345.86343	4474.239748	3.0612	2.23E-11	3.16E-10	AOL_s00215g615	36h
22891708	24324.86871	5047.656985	2.2687	3.77E-08	2.89E-07	AOL_s00075g198	

Supplementary Table S3. List of RT-PCR primers used in this study.

MVB-related genes	Primer sequence
β-tubulin	5F-CCACCTICGTCGGTAACIC<br>3R-TCGTCCATACCCTCACCAG
AOL_S00054g506(vps4)	5F-GAGGATGTTGCTGGTTTG<br>3R-ACTGCTCACGGAGAAGAA
AOL_S00075g200(vps23)	5F-AGCATACGACGACGGACG<br>3R-GCCAGATAAGGGTGATAGCA
AOL_S00043g16(vps24)	5F-AGCAGCTCACCCGTCAAG<br>3R-CGACTCGCAAGTCTCAAAA
AOL_S00193g153(vps28)	5F-CCGAAGTTACAAATTGACG<br>3R-GTTCTCAGTCCCATACGC
AOL_S00054g689(snf7)	5F-ATCGCCTCTTCGGCACCA<br>3R-GGGACCGTCACGCATTTT

Supplementary Table S4. Models used in phylogenetic tree analysis.

Model	#Param	BIC	AICc	lnL	Invariant	Gamma
**LG+G**	**22**	**11039.6604**	**10900.5143**	**-5428.135187**	**n/a**	**1.34929717**
LG+G+I	23	11047.9965	10902.5367	-5428.135252	0.00001	1.34820892
WAG+G	22	11078.8323	10939.6862	-5447.721115	n/a	1.60381071
JTT+G	22	11085.6377	10946.4916	-5451.123836	n/a	1.49422991
WAG+G+I	23	11087.1694	10941.7096	-5447.721705	0.00001	1.60481928
JTT+G+I	23	11093.9744	10948.5146	-5451.124201	0.00001	1.49490304
LG+I	22	11101.7516	10962.6055	-5459.180766	0.05456446	n/a
rtREV+G	22	11105.4841	10966.338	-5461.04703	n/a	1.4051738
rtREV+G+I	23	11113.8198	10968.3601	-5461.046943	0	1.40434131
WAG+I	22	11129.6703	10990.5242	-5473.140106	0.05121076	n/a
Dayhoff+G	22	11130.4654	10991.3194	-5473.537701	n/a	1.46840727
LG+G+F	41	11131.8882	10872.95	-5395.057935	n/a	1.33345822
LG	21	11134.8049	11001.9735	-5479.875378	n/a	n/a
JTT+I	22	11135.5151	10996.369	-5476.062533	0.05494535	n/a
Dayhoff+G+I	23	11138.8015	10993.3418	-5473.53778	0	1.46892106
LG+G+I+F	42	11140.2266	10874.9933	-5395.059157	0.00001	1.33419502
WAG	21	11154.1809	11021.3495	-5489.563391	n/a	n/a
rtREV+G+F	41	11156.8503	10897.9121	-5407.538977	n/a	1.31829754
rtREV+G+I+F	42	11165.1874	10899.9541	-5407.539563	0.00001	1.3182915

Supplementary Table S5. List of Gene knockout related primers in this study.

Primers	Sequence (5′-3′)	Description
Rvs167-5F	GTAACGCCAGGGTTTTCCCAGTCACGACGGCTACACCAGCCTTTTAT	*AoRvs167* 5′ flank
Rvs167-5R	ATCCACTTAACGTTACTGAAATCTCCAACGATTGCTTCGTATTCCTC	
Rvs167-3F	CTCCTTCAATATCATCTTCTGTCTCCGACTTTGACCTTACGCTGGAC	*AoRvs167* 3′ flank
Rvs167-3R	GCGGATAACAATTTCACACAGGAAACAGCCAATGAGACTGGCGATGT	
hph-F	GTCGGAGACAGAAGATGATATTGAAGGAGC	
hph-R	GTTGGAGATTTCAGTAACGTTAAGTGGAT	
Rvs167-F	TCACCCGTCCACCCTCTA	Identify positive transformant
Rvs167-R	CTTCCTCCGCTTTCTTCC	
RT_F	CCCATATCCGGTCGTATGTC	Identify mutant
RT_R	GGTTCTGTGCCTGTCGAAAT	
β-tubulin-F	CCACCTTCGTCGGTAACTC	
β-tubulin-R	TCGTCCATACCCTCACCAG	

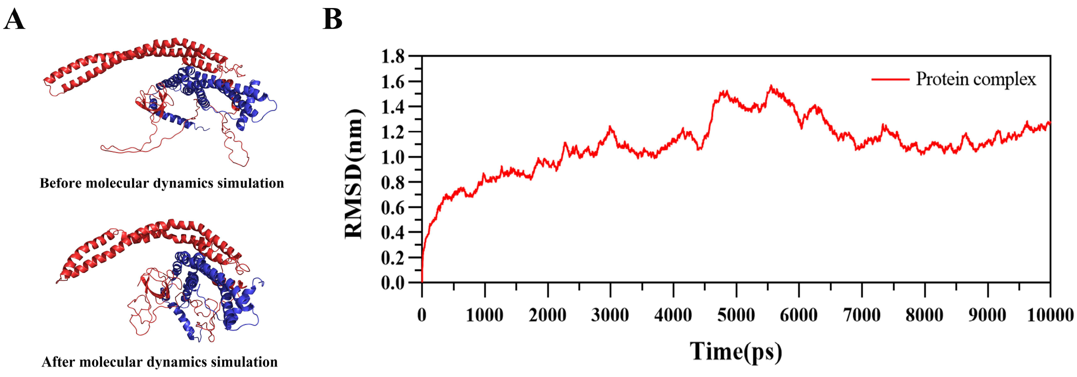

Supplementary Figure S1. Molecular dynamics simulations of the protein complexes were performed using Gromacs.(A) Changes in the structure of the complexe before and after molecular dynamics simulations; (B) RMSD value change during a 10 ns molecular dynamics simulation.

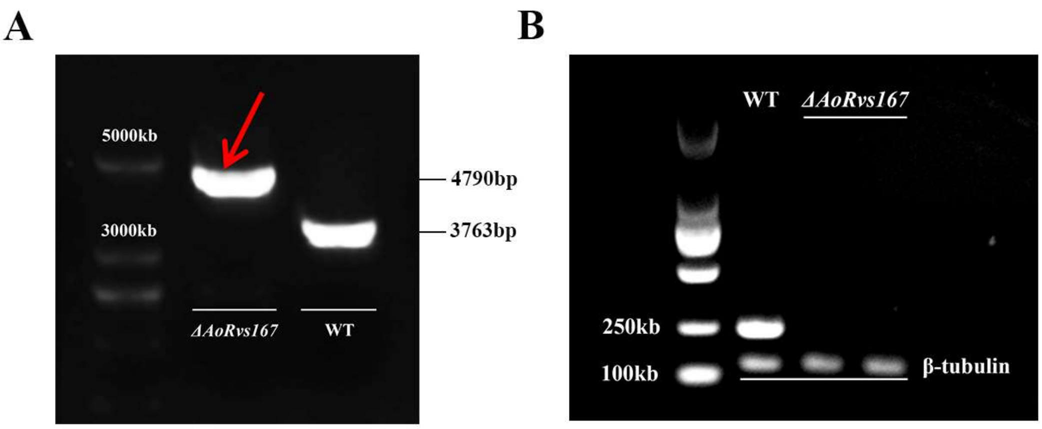

Supplementary Figure S2. PCR and RT-PCR.

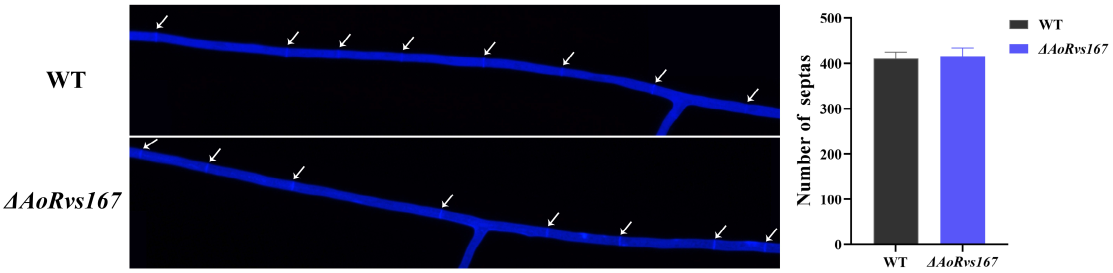

Supplementary Figure S3. Comparison of the hyphal septa between WT and *ΔAoRvs167*. The white arrow indicates hyphal septa (Bar=10μm).

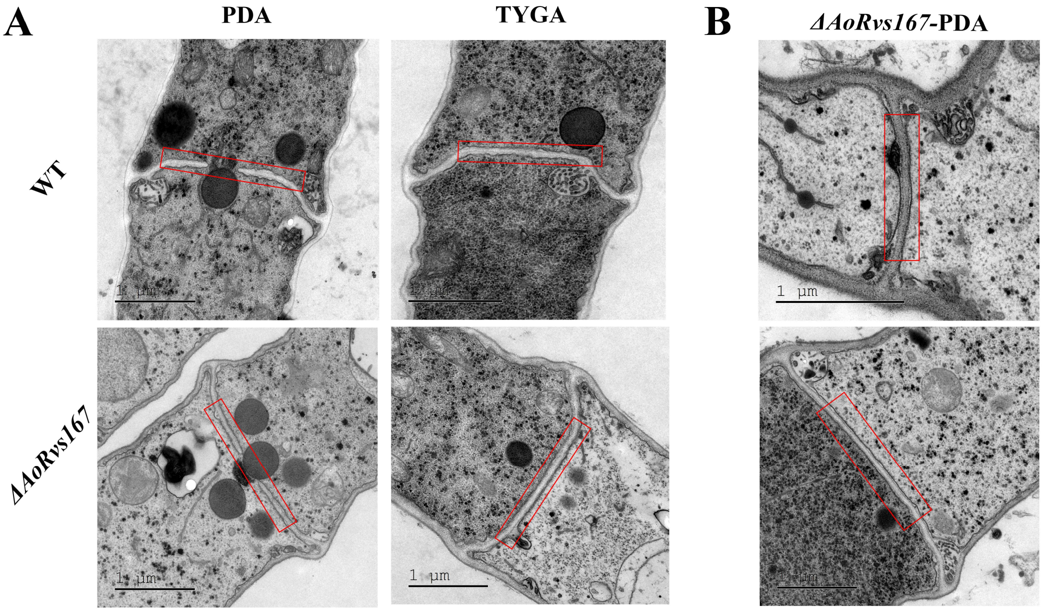

Supplementary Figure S4. TEM images further confirmed the conclusions drawn in Figure 3B. (A) Morphological differences of the WT and *ΔAoRvs167* membranes under different culture conditions observed under TEM (Bar=1μm). (B) is a supplement to (A), the membrane of *ΔAoRvs167* observed under TEM (Bar=1μm).

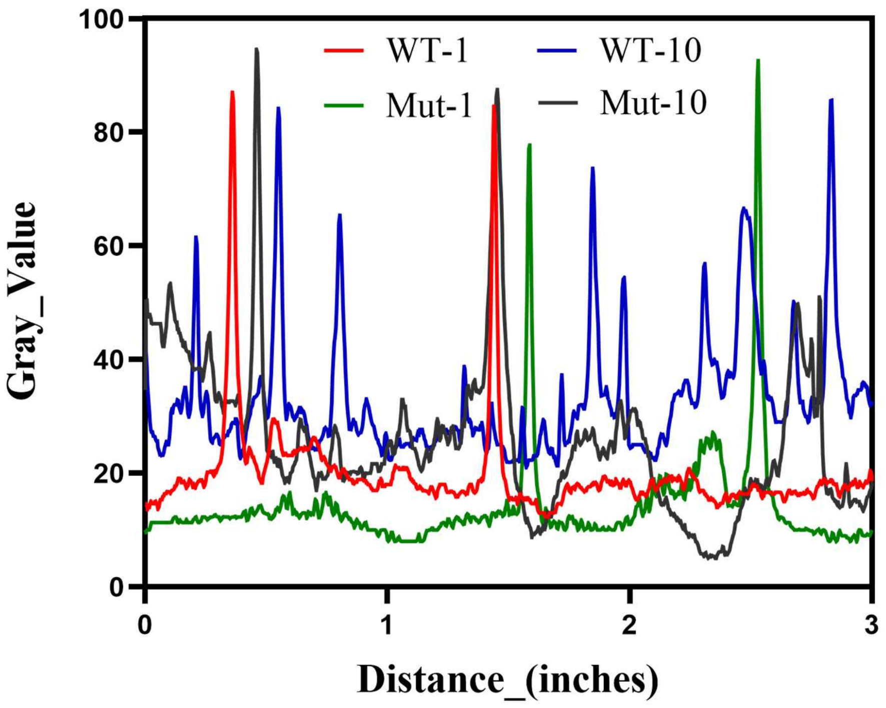

Supplementary Figure S5. Fluorescence intensity comparison between WT-1, WT-10, Mut-1, Mut-10 (WT-1 represents the WT strain stained for 1 min, Mut-1 represents the mutant strain stained for 1 min and so on).

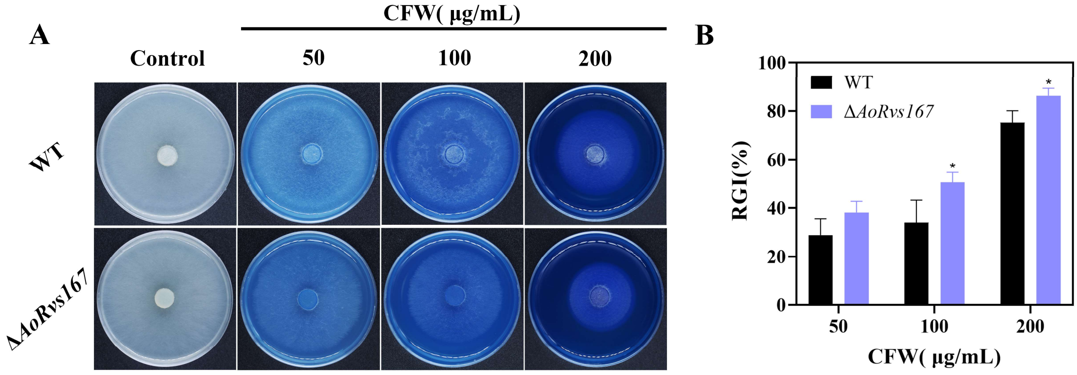

Supplementary Figure S6. Sensitivity of mutant strains to calcofluor white. (A) Colony area of WT and *ΔAoRvs167* strains cultured on PDA medium for 5 days at 25°C supplemented with different concentrations of calcofluor white (50-200 μg/mL). (B) Relative growth inhibition (RGI) was calculated.
